# Dorsomorphin Suppresses EMT to Reduce AR-Negative Prostate Cancer Metastasis by Synergistically Antagonizing JAK2/STAT3 and Gli2-Independent Shh Activation

**DOI:** 10.1155/proc/8843174

**Published:** 2025-09-30

**Authors:** Dongzhang Li, Guantao Lou, Wei Tian, Zujian Hu, Yongliang Chen, Wangjian Li

**Affiliations:** ^1^Department of Urology, Shaoxing Central Hospital, Shaoxing 312030, China; ^2^Key Laboratory of Diagnosis and Treatment of Severe Hepato-Pancreatic Diseases of Zhejiang Province, The First Affiliated Hospital of Wenzhou Medical University, Wenzhou 325000, China

**Keywords:** dorsomorphin, epithelial-mesenchymal transition (EMT), JAK2/STAT3, prostate cancer, sonic hedgehog, TGF-β1/Smad2/3

## Abstract

Prostate cancer is the most frequently diagnosed tumor of male reproductive system. Clinically, there is a lack of effective treatment drugs for prostate cancer. Previous studies have shown that AMPK inhibitor dorsomorphin was demonstrated to have potent antitumor effects. However, the effect of dorsomorphin on prostate cancer and its molecular mechanism are still unclear. In this study, the effects of dorsomorphin on the invasion and infiltration, epithelial-mesenchymal transition (EMT), and angiogenesis were investigated in two types of prostate cancer cells (DU145 and PC-3). In addition, nude mouse tumorigenic experiments were performed to confirm the antitumor effect of dorsomorphin. We found that dorsomorphin treatment concentration- and time-dependently inhibited the invasion and infiltration of DU145 and PC-3 cells. In addition, dorsomorphin reduced the expression levels of extracellular matrix components and angiogenesis-related proteins (HIF-1*α* and VEGF). Further study showed that dorsomorphin inhibited matrix deposition by antagonizing the EMT. Our results from nude mouse tumorigenic experiments further demonstrated dorsomorphin's tumor-growth inhibitory effect, whereas its antimetastatic potential is supported by in vitro invasion and EMT assays. Mechanistically, dorsomorphin treatment suppressed TGF-β1 expression and thereby inhibited the phosphorylation and nucleation of Smad2/3 signaling, which plays a key role in the regulation of EMT. Further study showed that dorsomorphin-triggered inactivation of JAK2/STAT3 and sonic hedgehog (Shh) signaling was involved in the inhibition of TGF-β1-mediated EMT. Interestingly, dorsomorphin inhibited the expression and nucleation of Gli1 and Gli3 but not affected the expression of Gli2. Thus, these findings reveal that the new mechanism of AMPK inhibitor dorsomorphin against prostate cancer metastasis is through synergistically antagonizing JAK2/STAT3 and Gli2-independent Shh activation.

## 1. Introduction

### 1.1. Prostate Cancer Overview

Prostate cancer is one of the most frequently malignant tumors of male reproductive system, accounting for about 10% of male malignant tumors [1]. The prostate is composed of parenchyma and stroma, and the parenchyma is composed of many acinar epithelial cells (AECs). When disordered growth of AECs occurs, that is, malignant hyperplasia occurs, prostate cancer will occur [2]. At present, the pathogenic factors of prostate cancer have not been fully clarified, which may be related to genetics, age, race, environment, food, obesity, and sex hormones. In addition, prostate cancer mainly occurs in elderly men, with an average age of 60–70 years. According to the condition of prostate cancer, surgery is the first choice, supplemented by radiotherapy, chemotherapy, and endocrine therapy [3]. The incidence of prostate cancer metastasis is relatively high, and the most common way is blood metastasis and lymph node metastasis [4]. Therefore, in-depth exploration of the intrinsic molecular mechanisms that lead to the metastasis of prostate cancer can help develop new strategies for treatment and drug selection in prostate cancer.

### 1.2. EMT and Metastasis

Studies have shown that in this type of refractory prostate cancer, there are often aberrant activations of multiple signaling pathways, including WNT/β-catenin, JAK2/STAT3, and sonic hedgehog (Shh)/Gli1/2/3 [5–7], resulting in an increase in the biological malignancy of tumor cells. Activated JAK2/STAT3, WNT/β-catenin, and Shh/Gli signaling induces the expression of epithelial-mesenchymal transition (EMT)-related transcription factors (EMT-TFs) such as Snail1 and Snail2 (Slug) through TGF-β1/Smad signaling, thus promoting EMT [8–11]. In the EMT process, epithelial cells are transformed to mesenchymal cells, and thereby cells have acquired the ability to transfer and immunosuppress. It not only plays a crucial role in organ development but also participates in the process of cancer evolution. In the process of cancer, the EMT is the key step to mediate the metastasis of epithelial-derived tumor cells. Several studies have also confirmed the role of EMT in the metastasis and drug resistance of prostate cancer. Blocking the key signal pathway and then reversing EMT may be one of the effective strategies to inhibit prostate cancer metastasis [12, 13].

### 1.3. Dorsomorphin Potential

Dorsomorphin, known as compound C or BML-275, is a selective ATP-competitive AMPK inhibitor [14, 15]. Dorsomorphin has been shown to play an antitumor role by inducing apoptosis and protective autophagy through the Akt/mTOR-independent pathway [16, 17]. However, the effect of dorsomorphin on metastatic tumors is not very clear. In pulmonary fibrosis, dorsomorphin treatment significantly inhibited alveolar EMT [18]. In AML12 cells, dorsomorphin suppressed TGF-beta1-induced Smad3 transcriptional activity and EMT [19].

### 1.4. Research Hypothesis

While dorsomorphin was initially characterized as an AMPK inhibitor, emerging evidence suggests its pleiotropic effects on multiple pathways including BMP and TGF-β signaling [1, 2]. Its multifaceted activity makes it particularly interesting for targeting complex metastatic networks in prostate cancer. Based on this, we speculate that dorsomorphin may also have the effect of antagonizing prostate cancer metastasis via EMT inhibition.

### 1.5. Experimental Design

In this study, the effects of dorsomorphin on prostate cancer cell (DU145 and PC-3) invasion and infiltration were investigated. In addition, EMT and angiogenesis of prostate cancer cells were analyzed after dorsomorphin treatment. Furthermore, nude mouse tumorigenic experiments were performed to confirm the antitumor effect of dorsomorphin. Taken together, our findings indicated that dorsomorphin inhibits EMT to reduce prostate cancer metastasis by synergistically antagonizing JAK2/STAT3 and Gli2-independent Shh activation.

## 2. Materials and Methods

### 2.1. Cell Culture and Drug Treatment

Human prostate cancer cell lines (DU145 and PC-3) were obtained from the Shanghai Chinese Academy of Sciences Cell Bank (Shanghai, China) and cultured in Dulbecco's Minimum Essential Medium (DMEM/F12; Invitrogen, Grand Island, NY, USA). The DMEM contained streptomycin (100 μg/mL, Invitrogen), penicillin (100 U/mL, Invitrogen), and fetal bovine serum (10% FBS, Invitrogen). The cells were treated with dorsomorphin (Cat. no. HY-13418A, MCE, Shanghai, China) in a 37°C-cell incubator with 5% CO_2_.

### 2.2. Wound Healing Assay

The wound healing assay was conducted to determine the mobility of DU145 and PC-3 cells. The cells were cultured in a six-well plate at a cell number of 3 × 10^5^ cells/well. When the cells grew and were more than 95% confluent, the cells were scraped off with 200-μL pipette tips to form a strip about 1-mm wide. Cell-free areas were washed with sterile phosphate-buffered saline (PBS) to remove exfoliated cells, and pictures were obtained under a microscope once the scratches were just formed. Finally, the culture medium without FBS and corresponding drugs were added to continue the culture. Photographs were taken every 24 h. Experiments were independently performed at least in triplicate.

### 2.3. Transwell Analysis

Transwell analysis was used to evaluate the invasive ability of DU145 and PC-3 cells. In brief, the medium without FBS was used to resuspend prostate cancer cells to be added to the precoated Transwell chamber (4 × 10^4^ cells/well, Corning Costar, NY, USA). Furthermore, the medium (600 μL) containing 15% FBS and the corresponding drugs were added to the lower and the upper chamber, respectively. After treatment for 24 h, cells were fixed with 4% paraformaldehyde (Solarbio) and stained with crystal violet (Solarbio). Finally, the cells in the Transwell chamber were wiped off and images of invaded cells were obtained under a microscope (Leica Microsystems, Wetzlar, Germany). All experiments were performed independently at least in triplicate.

### 2.4. Immunoblotting Assay

Immunoblotting assay was performed according to a previously reported protocol [20, 21]. In brief, the cells were treated with dorsomorphin in 10-cm Petri dishes for 24 h, and the total protein was collected using M-PER mammalian protein extraction reagent (Thermo Scientific). Then, The concentration of the total protein was analyzed using a BCA (bicinchoninic acid) protein concentration assay kit (GLPBIO). The proteins were denatured at 100°C and electrophoresed via 10% SDS-PAGE gels. The electrophoretically separated proteins were then transferred to polyvinylidene fluoride (PVDF) membranes (Sigma-Aldrich, Shanghai, China) and then were blocked with 5% skimmed milk for 2 h before incubation with primary antibodies: GAPDH (internal reference), Col-1, Col-3, Snail1, Slug, E-cadherin, N-cadherin, *α*-SMA, vimentin, p-Smad2, p-Smad3, Smad2/3, TGF-β1, HIF-1*α*, VEGF, JAK2, p-JAK2, STAT3, p-STAT3, WNT, *β*-catenin, p-β-catenin, Shh, Gli1, Gli2, and Gli3 at 4°C overnight. Subsequently, PVDF membrane was treated with horseradish peroxidase (HRP)-conjugated secondary antibodies (ProteinTech, Wuhan, China), and western blot results were visualized using the SuperSignal West Pico Chemiluminescent Substrate device (Thermo Fisher Scientific). Finally, the grayscale values were analyzed using the ImageJ software (NIH, Bethesda, MD, USA).

### 2.5. Immunofluorescence Staining

Cells were treated with dorsomorphin for 24 h and then fixed with 4% paraformaldehyde (Solarbio). Subsequently, 0.1% Triton ×100 (Solarbio) and normal goat serum (BOSTER, Wuhan, China) were used for membrane permeation blocking and nonspecific staining, respectively. Next, the primary antibodies, including Col-3, *α*-SMA, Snail1, p-Smad3, HIF-1*α*, p-STAT3, Gli1, Gli2, and Gli3, were added to incubate at 4°C overnight. Next, the secondary antibodies DyLight 594-red or 488-green fluorescence (ProteinTech) were allowed to bind to primary antibodies. Finally, DAPI (Solarbio) was used to stain cell nuclei and a fluorescence microscope (Leica) was used to take images.

### 2.6. Nude Mouse Tumorigenicity Experiments

BALB/c nude mice (*n* = 12, 18–22 g) from Wenzhou Medical University were housed in a humidity-, temperature-, and light-controlled environment. The mice were fed standard chow and water. First, 100 μL of cell suspension (5 × 10^6^ PC-3 cells) were injected subcutaneously into the abdomen of each nude mouse. Each mouse that successfully formed a tumor (2-mm diameter) was randomly assigned to two groups using a computer-generated randomization sequence (Research Randomizer v4.0) with researchers blinded to group allocation during data collection and analysis. Mice received daily intraperitoneal injections of dorsomorphin (10 mg/kg, *n* = 6) or solvent (saline, *n* = 6) consecutively for 4 weeks. The mice were anesthetized at a dose of 1%–3% inhalation anesthetic for 5–15 min. The weight of the nude mice was recorded 1 week after prostate cancer cell injection. The mice were euthanized when the tumor reached a maximum volume of 1000 mm^3^ or other humanitarian endpoints (such as 20% weight loss, severe infections, and ulcers). Only two mice developed ulcers, and as soon as the ulcers developed, they were euthanized on humanitarian grounds by continuous CO_2_ inhalation. Complete cessation of respiration and heartbeat in mice is considered mouse death. The animal study protocols including the procedure of animals' euthanasia were approved by the Institutional Animal Care and Use Committee of Wenzhou Medical University (WYYY-AEC-2022-041).

### 2.7. Immunohistochemical (IHC) Staining

Tissues fixed in 4% paraformaldehyde (Solarbio) were paraffinized and sliced into 4-μm thick sections. The sections were dehydrated across an ethanol gradient (100%, 95%, 85%, and 75%). After goat serum (25 μL, BOSTER) treatment to inactivate endogenous peroxidase, antigen retrieval was performed in the sections containing 0.1% sodium citrate buffer (pH = 6.0). Then, the sections were incubated with primary antibodies overnight and secondary antibodies (ProteinTech) with HRP for 1 h, respectively. Finally, the DAB HRP Color Kit was used to determine the protein expression and location. The IHC samples were semiquantitatively evaluated by two independent researchers who were blinded to group allocation.

### 2.8. Statistical Analysis

The results are presented as the mean ± standard deviation (SD) using the Image J software (Version 1.4.3, NIH, USA) or a GraphPad Prism software (Version 8.3.0, GraphPad Software, USA). Two-sided Student's *t*-test and one-way analysis of variance (ANOVA) were performed for statistical analysis when there were two experimental groups or more than two experimental groups, respectively. A *p* value less than 0.05 is considered statistically significant.

All quantitative experiments including Western blotting and functional assays were performed with a minimum of three biological replicates. Sample sizes for animal studies were determined based on our previous xenograft experiments with similar effect sizes.

## 3. Results

### 3.1. Dorsomorphin Reduces Prostate Cancer Cell Migration and Invasion

**Figure 1:**
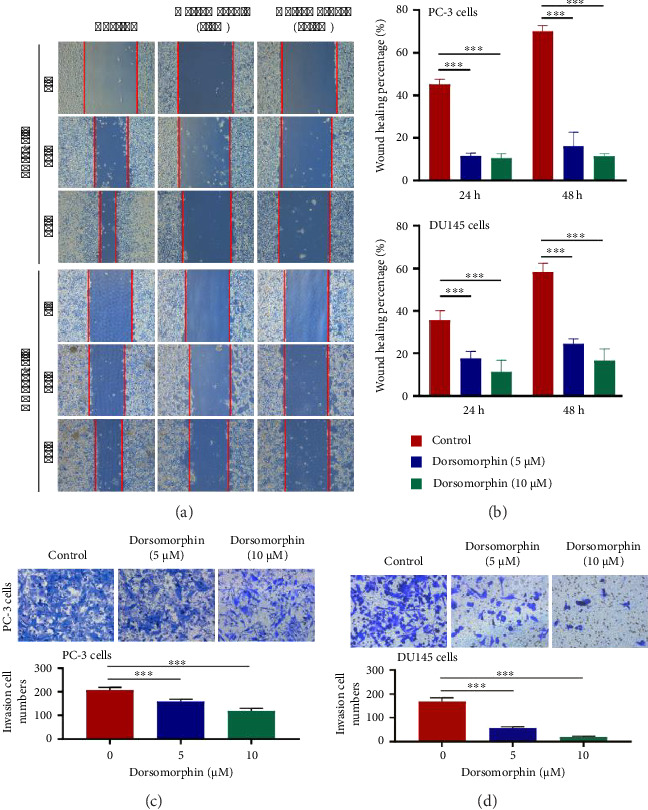
Effects of dorsomorphin on the invasion and infiltration of prostate cancer cells. (a-b) The migration rate of dorsomorphin-treated PC-3 and DU145 cells as determined by wound healing assay. (c-d) The invasion number of dorsomorphin-treated PC-3 and DU145 cells analyzed by the Transwell chamber assay. Data are presented as the mean ± standard deviation in quintuplicate for the cell line experiment. ^∗∗∗^*p* < 0.001. Data are presented as the mean ± SD from three independent experiments. ^∗∗∗^*p* < 0.001, ^∗∗^*p* < 0.01, and ^∗^*p* < 0.05 by one-way ANOVA with Tukey's post hoc test.

Tumor cell metastasis is the most important factor associated with the poor prognosis of prostate cancer patients. In the present study, dorsomorphin treatment concentration- and time-dependently decreased the migration rate of PC-3 cells, as determined by the results of the wound healing assay (Figures 1(a) and 1(b)). Also, dorsomorphin concentration- and time-dependently reduced the migration rate of DU145 cells (Figures 1(a) and 1(a)). In addition, the invasion number of PC-3 and DU145 cells in the Transwell chamber assay was significantly suppressed by dorsomorphin (Figures 1(c) and 1(d)). Thus, these findings indicated that dorsomorphin has an inhibitory effect on prostate cancer cell migration and invasion.

### 3.2. Dorsomorphin Reduces Matrix Deposition and VEGF Expression in Prostate Cancer Cells

**Figure 2:**
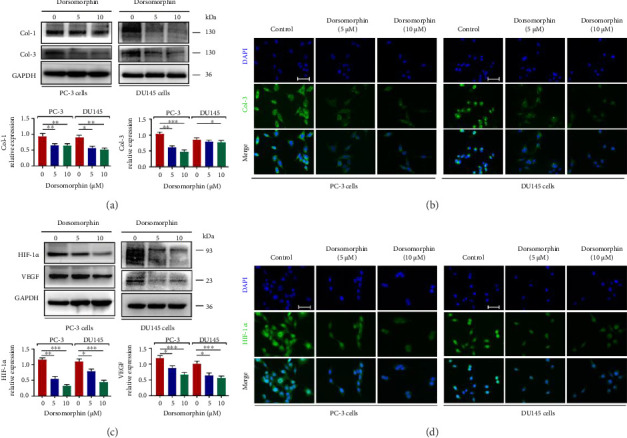
Effects of dorsomorphin on matrix deposition, HIF-1*α*, and VEGF expression of prostate cancer cells. (a) Western blot analysis showing protein expressions of Col-1 and Col-3 in dorsomorphin-treated PC-3 and DU145 cells. (b) Immunocytochemical staining of Col-3 in dorsomorphin-treated PC-3 and DU145 cells. Bar = 25 μM. (c) Western blot analysis showing protein expressions of HIF-1*α* and VEGF in dorsomorphin-treated PC-3 and DU145 cells. (d) Immunocytochemical staining of HIF-1*α* in dorsomorphin-treated PC-3 and DU145 cells. Bar = 25 μM. Data are presented as mean ± standard deviation in quintuplicate for the cell line experiment. ^∗^*p* < 0.05; ^∗∗^*p* < 0.01; ^∗∗∗^*p* < 0.001. Data are presented as the mean ± SD from three independent experiments. ^∗∗∗^*p* < 0.001, ^∗∗^*p* < 0.01, and ^∗^*p* < 0.05 by one-way ANOVA with Tukey's post hoc test.

Next, we investigated the effects of dorsomorphin on extracellular matrix deposition and VEGF expression in prostate cancer cells. As expected, dorsomorphin reduced the expression of type I and III collagens (Figures 2(a) and 2(b)), the main components in the extracellular matrix that drive tumor tissue substantiation. Also, dorsomorphin decreased the expression of HIF-1*α* and VEGF (Figures 2(c) and 2(d)). HIF-1*α* and its downstream VEGF play important roles in tumor angiogenesis [22]. Downregulated expression of HIF-1*α* and VEGF indicates an antiangiogenic effect of dorsomorphin in prostate cancer cells. Thus, these results showed that dorsomorphin inhibits matrix deposition and antagonizes the expression of HIF-1*α* and VEGF to hinder angiogenesis.

### 3.3. Dorsomorphin Inhibits the EMT Process In Vitro and In Vivo

**Figure 3:**
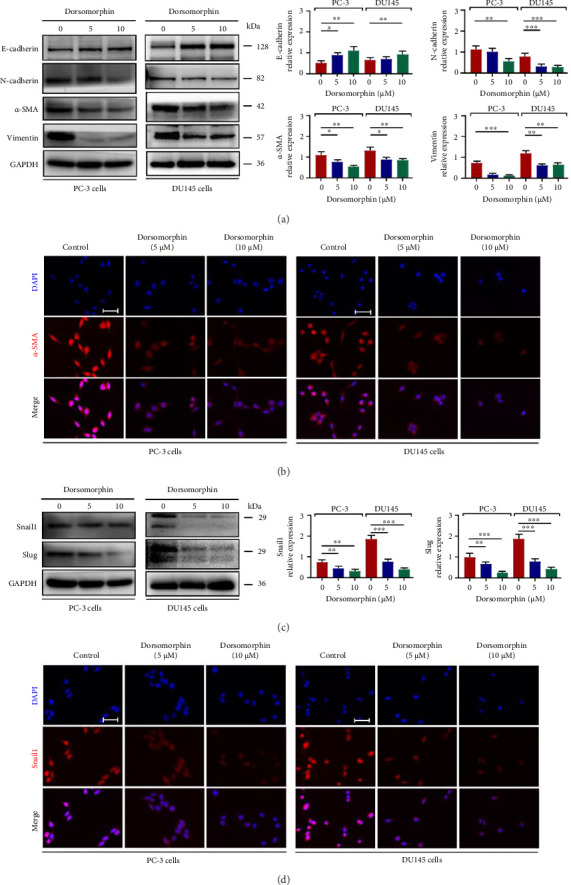
Effects of dorsomorphin on the EMT process in prostate cancer cells. (a) Western blot analysis showing the protein expression of E-cadherin, N-cadherin, *α*-SMA, and vimentin in dorsomorphin-treated PC-3 and DU145 cells. (b) Immunocytochemical staining of *α*-SMA in dorsomorphin-treated PC-3 and DU145 cells. Bar = 25 μM. (c) Western blot analysis showing the protein expressions of Snail1 and Slug in dorsomorphin-treated PC-3 and DU145 cells. (d) Immunocytochemical staining of Snail1 in dorsomorphin-treated PC-3 and DU145 cells. Bar = 25 μM. Data are presented as the mean ± standard deviation in quintuplicate for the cell line experiment. ^∗^*p* < 0.05; ^∗∗^*p* < 0.01; and ^∗∗∗^*p* < 0.001. Data are presented as the mean ± SD from three independent experiments. ^∗∗∗^*p* < 0.001, ^∗∗^*p* < 0.01, and ^∗^*p* < 0.05 by one-way ANOVA with Tukey's post hoc test.

**Figure 4:**
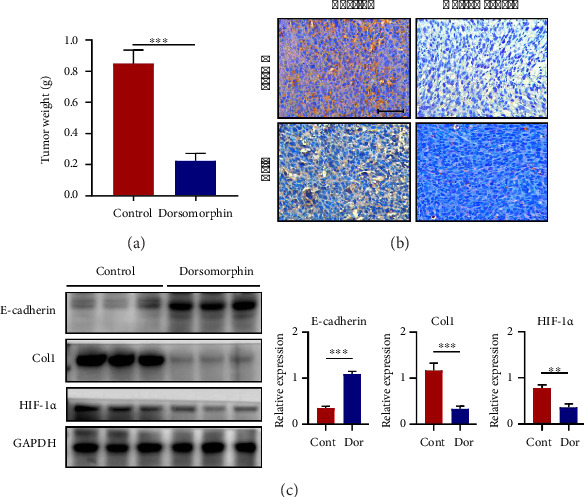
Effects of dorsomorphin on tumor growth and EMT in a nude mouse tumorigenic model. (a) Effects of dorsomorphin on tumor weight in the animal xenograft model. (b) IHC staining for *α*-SMA and Col1 in the dorsomorphin-treated model. Bar = 25 μM. (c) Western blot analysis showing protein expressions of E-cadherin, Col1, and HIF-1*α* in the dorsomorphin-treated model. Data are presented as the mean ± standard deviation in quintuplicate for the animal experiment. ^∗^*p* < 0.05; ^∗∗^*p* < 0.01; and ^∗∗∗^*p* < 0.001. Data are presented as the mean ± SD from three independent experiments. ^∗∗∗^*p* < 0.001, ^∗∗^*p* < 0.01, and ^∗^*p* < 0.05 by one-way ANOVA with Tukey's post hoc test.

EMT is an important process by which epithelial-derived cancer cells acquire the ability to migrate and invade [12, 13]. In this study, the protein expressions of N-cadherin, vimentin, and *α*-SMA were decreased, whereas the expression of E-cadherin was increased following dorsomorphin treatment in DU145 and PC-3 cells (Figures 3(a) and 3(b)). In addition, dorsomorphin inhibited the expression levels of EMT-TFs, including Snail1 and Slug (Figures 3(c) and 3(d)). In prostate cancer xenograft nude mice in vivo, dorsomorphin treatment (10 mg/kg·d) reduced tumor weight (Figure 4(a)). Also, dorsomorphin downregulated the expression of collagen I, *α*-SMA, and HIF-1*α* and upregulated the expression of E-cadherin (Figures 4(b) and 4(c)). Thus, these findings confirmed again that dorsomorphin inhibits the EMT process in prostate cancer cells. These in vivo findings corroborate dorsomorphin's growth-suppressive activity; conclusions regarding metastasis suppression derive from in vitro invasion and EMT analyses.

### 3.4. Dorsomorphin Inhibits TGF-β1/Smad2/3 Signaling Activation in Prostate Cancer Cells

**Figure 5:**
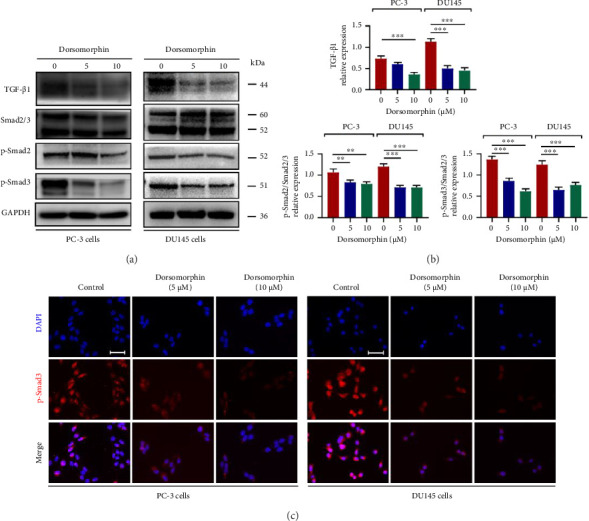
Effects of dorsomorphin on TGF-β1/Smad2/3 signaling activity in prostate cancer cells. (a-b) Western blot analysis showing protein expressions or phosphorylation of TGF-β, Smad2, and Smad3 in dorsomorphin-treated PC-3 and DU145 cells. (c) Immunocytochemical staining of p-Smad3 in dorsomorphin-treated PC-3 and DU145 cells. Bar = 25 μM. Data are presented as the mean ± standard deviation in quintuplicate for the cell line experiment. ^∗∗^*p* < 0.01 and ^∗∗∗^*p* < 0.001. Data are presented as the mean ± SD from three independent experiments. ^∗∗∗^*p* < 0.001, ^∗∗^*p* < 0.01, and ^∗^*p* < 0.05 by one-way ANOVA with Tukey's post hoc test.

Numerous studies have shown a key role of TGF-β1/Smad signaling in EMT induction [8]. We found that dorsomorphin treatment inhibited TGF-β1 expression (Figure 5(a)) and downregulated the phosphorylation of TGF-β1-downstream cascade Smad2 and Smad3 (Figures 5(a) and 5(b)). As a result, nuclear expression of Smad3 was inhibited by dorsomorphin (Figure 5(c)). Thus, these results suggested that dorsomorphin reduces TGF-β1/Smad2/3 activity to hinder EMT in prostate cancer cells.

### 3.5. Dorsomorphin Antagonizes the Activation of JAK2/STAT3 and Gli2-Independent Shh Signaling

**Figure 6:**
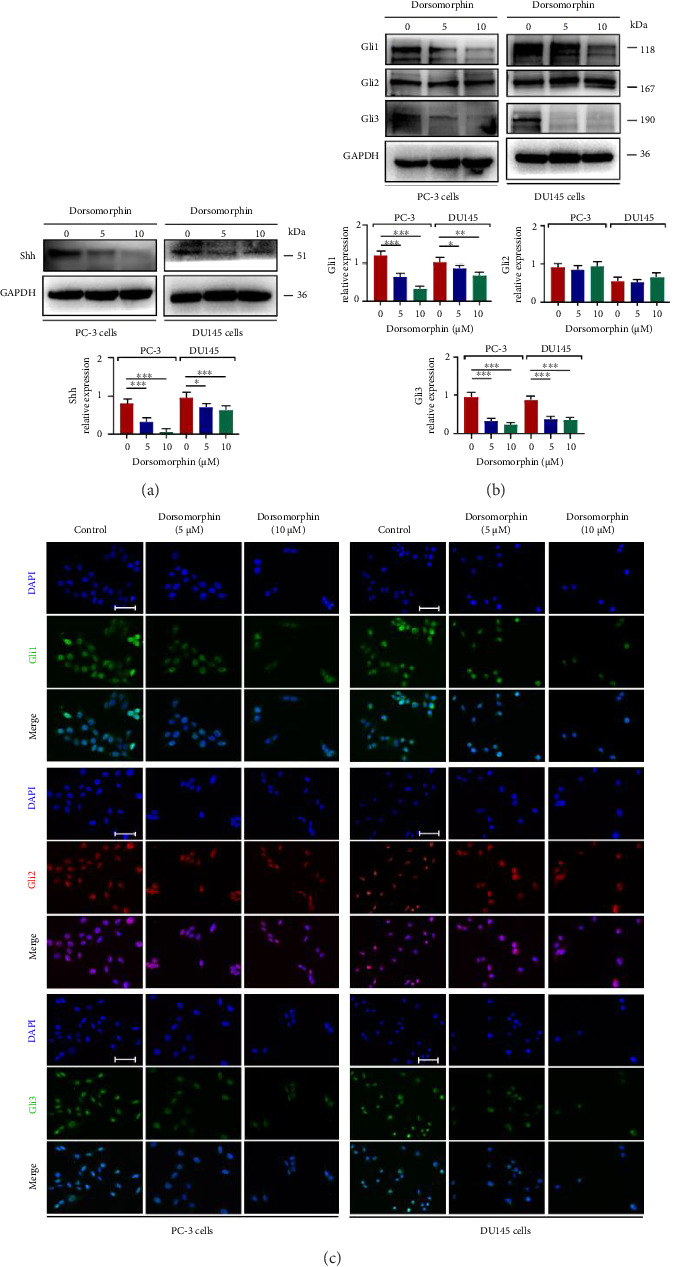
Effects of dorsomorphin on the activation of Shh/Gli signaling in prostate cancer cells. (a) Western blot analysis showing protein expression of Shh in dorsomorphin-treated PC-3 and DU145 cells. (b) Western blot analysis showing protein expression of Gli1, Gli2, and Gli3 in dorsomorphin-treated PC-3 and DU145 cells. (c) Immunocytochemical staining of Gli1, Gli2, and Gli3 in dorsomorphin-treated PC-3 and DU145 cells. Bar = 25 μM. Data were presented as the mean ± standard deviation in quintuplicate for the cell line experiment. ^∗^*p* < 0.05; ^∗∗^*p* < 0.01; and ^∗∗∗^*p* < 0.001. Data are presented as the mean ± SD from three independent experiments. ^∗∗∗^*p* < 0.001, ^∗∗^*p* < 0.01, and ^∗^*p* < 0.05 by one-way ANOVA with Tukey's post hoc test.

**Figure 7:**
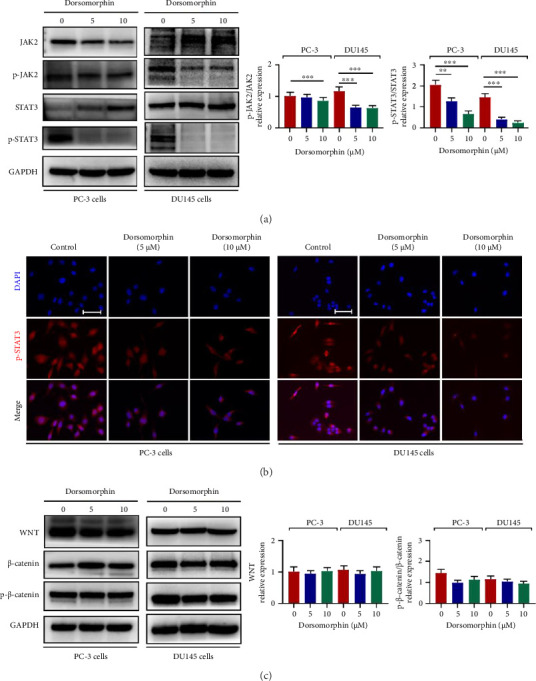
Effects of dorsomorphin on the activation of JAK2/STAT3 and WNT/β-catenin signaling pathways in prostate cancer cells. (a) Western blot analysis showing protein expression or phosphorylation of JAK2 and STAT3 in dorsomorphin-treated PC-3 and DU145 cells. (b) Immunocytochemical staining of p-STAT3 in dorsomorphin-treated PC-3 and DU145 cells. Bar = 25 μM. (c) Western blot analysis showing protein expression or phosphorylation of WNT and *β*-Catenin in dorsomorphin-treated PC-3 and DU145 cells. Data were presented as the mean ± standard deviation in quintuplicate for the cell line experiment. ^∗∗^*p* < 0.01 and ^∗∗∗^*p* < 0.001. Data are presented as the mean ± SD from three independent experiments. ^∗∗∗^*p* < 0.001, ^∗∗^*p* < 0.01, and ^∗^*p* < 0.05 by one-way ANOVA with Tukey's post hoc test.

Notably, while dorsomorphin significantly downregulated Gli1 and Gli3 expression (*p* < 0.01), it exhibited no inhibitory effect on Gli2 (*p* > 0.05; Figure 6(b)), suggesting a distinct regulatory mechanism for this transcription factor in AR-negative cells [3]. To further elucidate the molecular mechanism underlying the antiprostate cancer effect of dorsomorphin, we examined the activities of Shh/Gli1/2/3, WNT/β-catenin, and JAK2/STAT3 pathways, which involves in the regulation of TGF-β1/Smad signaling. First, dorsomorphin inhibited the phosphorylation and nuclear expression of JAK2 and STAT3 in DU145 and PC-3 cells, suggesting that dorsomorphin antagonized the activation of JAK2/STAT3 signaling pathway (Figures 7(a) and 7(b)). However, dorsomorphin did not affect the activity of WNT/β-catenin signaling in DU145 and PC-3 cells (Figure 7(c)). In addition, dorsomorphin treatment decreased the expression of Shh, a ligand for Shh signaling (Figure 6(a)). Interestingly, dorsomorphin inhibited the expression of hedgehog-downstream transcription factors Gli1 and Gli3 in DU145 and PC-3 cells but did not downregulate the expression of Gli2 (Figures 6(b) and 6(c)), indicating that there may be some differences between the two types of prostate cancer cells in the regulation mechanism of dorsomorphin on Shh signaling. Taken together, dorsomorphin exerts antitumor effects in prostate cancer cells by synergistically antagonizing the activation of JAK2/STAT3 and Gli2-independent Shh pathways.

## 4. Discussion

Here, we investigated the antitumor effects of dorsomorphin in prostate cancer in vivo and in vitro. We found that dorsomorphin treatment concentration- and time-dependently inhibited the invasion and infiltration of PC-3 and DU145 cells. In addition, dorsomorphin inhibited matrix deposition and antagonized the expression of HIF-1*α* and VEGF. Furthermore, dorsomorphin treatment (10 mg/kg·d) reduced tumor weight in a prostate cancer xenograft nude mouse. Further study showed that the dorsomorphin-mediated inhibition of EMT may contribute to the antitumor effects in prostate cancer. Thus, our experiments indicated that dorsomorphin inhibits the EMT process to reduce prostate cancer metastasis.

Three key limitations should be noted: first, the exclusive use of AR-negative models, while clinically relevant for castration-resistant disease, precludes generalization to AR-positive prostate cancer. Second, although we demonstrate dorsomorphin's efficacy in suppressing metastasis-related pathways, whether the observed effects are mediated by AMPK inhibition or by off-target modulation of BMPR, TGF-βR1, or mitochondrial complex I (Hawley et al., 2022) requires CRISPR-mediated AMPKα1/*α*2 knockout or kinase-dead knock-in experiments. Third, the intriguing Gli2-independent mechanism warrants deeper investigation through overexpression/knockdown studies.

EMT is an important factor promoting tumor metastasis [9, 13]. In prostate cancer, EMT is regulated by EMT-TFs dependent of TGF-β1/Smad signaling activation [24]. A series of evidences showed that EMT-TFs are involved in regulation of the EMT process from the transcriptional level, post-transcriptional level, and translation. For example, Snail1 can inhibit the expression of E-cadherin gene by blocking an E-box upstream of the gene [25, 26]. In addition, Snail1 can also interact with enhancer of zeste homolog 2 (EZH2), G9a, SUV39H1, and other methyl transfer enhancer to form a ploycomb repressive complex2 (PRC2) to jointly regulate the EMT process [27, 28]. Further studies showed that the expression of EMT-TFs is regulated by TGF-β1/Smad2/3 signaling. After TGF-β1 stimulation, Smad2 and Smad3 can be phosphorylated and form a polymer with Smad4, which can bind to target genes and regulate the expression of EMT-TFs Snail1 and Slug [29]. In our study, dorsomorphin treatment significantly reduced the expression levels of Snail1 and Slug. In addition, dorsomorphin inhibited TGF-β1 expression and downregulated the phosphorylation and nuclear expression of TGF-β1-downstream cascade Smad2 and Smad3. It is important to emphasize that the xenograft model used here measures primary tumor growth rather than spontaneous metastasis; thus, antimetastatic claims are based on in vitro assays. Thus, dorsomorphin hindered the EMT process by antagonizing TGF-β1/Smad2/3 signaling activation and subsequent EMT-TF expression.

Further study showed that the inhibitory effect of dorsomorphin on TGF-β1/Smad2/3-mediated EMT was associated with the inactivation of JAK2/STAT3 and Shh signaling. These signaling pathways play a crucial role in the occurrence and development of prostate cancer by regulating the release of proinflammatory cytokines and affecting cell proliferation, apoptosis, tumor metabolism, genomic stability, and tumor drug resistance [7, 8, 10]. In addition, these pathways were involved in the regulation of EMT and VEGR-mediated angiogenesis in prostate cancer [10, 30, 31]. The activation of these signaling pathways can interact with TGF-β1/Smad signaling, thereby promoting the EMT and triggering tumor cell invasion and infiltration. This interaction is manifested not only in regulating the expression level of TGF-β1 but also by regulating Smad3 phosphorylation, thereby participating in its signal transduction pathway. In addition, these signaling pathways themselves can directly regulate the expression of EMT-TFs, thereby affecting the EMT process [7, 8, 10]. Therefore, the inhibitory effect of dorsomorphin on JAK2/STAT and Shh signaling in this study may to some extent be one of the mechanisms by which dorsomorphin antagonizes EMT and prostate cancer cell invasion and infiltration.

Interestingly, dorsomorphin inhibited the expression of hedgehog-downstream transcription factors Gli1 and Gli3 in DU145 and PC-3 cells but did not affect the expression of Gli2. Previous studies have shown that different Gli proteins exhibit different biological functions [32]. For example, Gli2 is a strong gene activator, but due to its N-terminal containing an inhibitory region, once the protein undergoes degradation, Gli2 may exhibit transcriptional inhibitory activity [33]. In the hypoxic microenvironment of tumors, Gli2 can be activated to regulate HIF-1 *α* and TGF-β expression and induce chemotherapy resistance in colorectal tumors [34]. However, the mechanism of action of Gli2 in prostate cancer is unclear. We are currently unclear about the specific mechanism by which dorsomorphin inhibits Shh signaling activation through a Gli2 independent mode. We speculate that this may be related to the special structure of Gli2 protein.

There are some limitations in this study. First, the genetic approach in terms of the association between multiple signaling pathways in dorsomorphin-treated prostate cancer cells needs to be evaluated. In addition, the crosstalk between TGF-β/Smad2/3 and other signaling pathways needs to be studied. Importantly, the precise target of the antitumor effect of dorsomorphin should be further clarified and confirmed by pharmacological studies.

Dorsomorphin's multipathway inhibition (JAK2/STAT3 and Gli2-independent Shh) suggests potential synergy with existing mCRPC regimens such as taxane chemotherapy or AR-pathway inhibitors. For instance, combining dorsomorphin with docetaxel may counteract chemotherapy-induced EMT. Patient selection could leverage AR-negative or neuroendocrine biomarkers (e.g., low AR/PSA and high SYP/CHGA) to enrich for responders. Regarding dosing, the 10-mg/kg intraperitoneal dose in mice yields free plasma Cmax ≈ 2-3 μM [14], which is above the 0.5–1 μM required for EMT inhibition in vitro; this concentration is achievable in humans using existing AMPK-inhibitor formulations, although formal PK/PD studies are needed.

In conclusion, dorsomorphin inhibited the invasion and infiltration of prostate cancer cells by blocking the EMT process. In addition, dorsomorphin inhibited tumor angiogenesis by reducing the expression levels of HIF-1*α* and VEGF. Moreover, dorsomorphin inhibited tumor growth, EMT, and matrix deposition in a nude mouse model. Mechanistically, dorsomorphin may exert its antitumor effect in prostate cancer cells through antagonizing the activation of TGF-β/Smad signaling. Further study showed that the inactivation of JAK2/STAT3 and Gli2-independent Shh signaling contributed to dorsomorphin-mediated inhibition of TGF-β/Smad signaling and subsequent EMT blockade. Thus, dorsomorphin may be a potential candidate for the treatment of prostate cancer.

## Data Availability

Data used to support the findings of this study will be made available from the corresponding author upon reasonable request.
